# Estimation of the Transmission Risk of the 2019-nCoV and Its Implication for Public Health Interventions

**DOI:** 10.3390/jcm9020462

**Published:** 2020-02-07

**Authors:** Biao Tang, Xia Wang, Qian Li, Nicola Luigi Bragazzi, Sanyi Tang, Yanni Xiao, Jianhong Wu

**Affiliations:** 1 The Interdisciplinary Research Center for Mathematics and Life Sciences, Xi’an Jiaotong University, Xi’an 710049, China; btang66@yorku.ca (B.T.); yxiao@mail.xjtu.edu.cn (Y.X.); 2 Laboratory for Industrial and Applied Mathematics, Department of Mathematics and Statistics, York University, Toronto, ON M3J 1P3, Canada; crystallee@stu.xjtu.edu.cn (Q.L.); robertobragazzi@gmail.com (N.L.B.); 3 School of Mathematics and Information Science, Shaanxi Normal University, Xi’an 710119, China; xiawang@snnu.edu.cn (X.W.); sanyitang219@hotmail.com (S.T.); 4 School of Mathematics and Statistics, Xi’an Jiaotong University, Xi’an 710049, China; 5 Fields-CQAM Laboratory of Mathematics for Public Health, York University, Toronto, ON M3J 1P3, Canada

**Keywords:** coronavirus, infection management and control, travel restriction, mathematical model, SEIR model

## Abstract

Since the emergence of the first cases in Wuhan, China, the novel coronavirus (2019-nCoV) infection has been quickly spreading out to other provinces and neighboring countries. Estimation of the basic reproduction number by means of mathematical modeling can be helpful for determining the potential and severity of an outbreak and providing critical information for identifying the type of disease interventions and intensity. A deterministic compartmental model was devised based on the clinical progression of the disease, epidemiological status of the individuals, and intervention measures. The estimations based on likelihood and model analysis show that the control reproduction number may be as high as 6.47 (95% CI 5.71–7.23). Sensitivity analyses show that interventions, such as intensive contact tracing followed by quarantine and isolation, can effectively reduce the control reproduction number and transmission risk, with the effect of travel restriction adopted by Wuhan on 2019-nCoV infection in Beijing being almost equivalent to increasing quarantine by a 100 thousand baseline value. It is essential to assess how the expensive, resource-intensive measures implemented by the Chinese authorities can contribute to the prevention and control of the 2019-nCoV infection, and how long they should be maintained. Under the most restrictive measures, the outbreak is expected to peak within two weeks (since 23 January 2020) with a significant low peak value. With travel restriction (no imported exposed individuals to Beijing), the number of infected individuals in seven days will decrease by 91.14% in Beijing, compared with the scenario of no travel restriction.

## 1. Introduction

Coronaviruses are enveloped, single-stranded, positive-sense RNA viruses belonging to the family of *Coronaviridae* [1]. They cause generally mild respiratory infections, even though they are occasionally lethal. Since their discovery and first characterization in 1965 [2], three major, large-scale outbreaks have occurred, caused by emerging, highly pathogenic coronaviruses, namely, the “Severe Acute Respiratory Syndrome” (SARS) outbreak in 2003 in mainland China [3], the “Middle East Respiratory Syndrome” (MERS) outbreak in 2012 in Saudi Arabia [4,5] and the MERS outbreak in 2015 in South Korea [6,7]. These outbreaks have resulted in more than 8000 and 2200 confirmed SARS and MERS cases, respectively [8].

Recently, a fourth coronavirus outbreak has occurred in Wuhan, the capital city of the Hubei province and the seventh largest city of People’s Republic of China [9,10,11]. 

Since 31 December 2019, when the Wuhan Municipal Health Commission reported 27 cases of viral pneumonia, including 7 critically ill cases, the pneumonia outbreak has received considerable global attention. A novel coronavirus was identified as the causative agent by the Chinese authorities on 7 January 2020, and on 10 January 2020, the World Health Organization (WHO) designated the novel coronavirus as 2019-nCoV. On the same day, the WHO released a wide range of interim guidance for all countries on how they can get prepared for coping with this emergency, including how to monitor for potentially infected people, collect and test samples, manage patients, control and mitigate the burden generated by the infection in health centers, maintain the right drug supplies and effectively communicate with the lay public regarding the new virus [12].

By the morning of 23 January 2020, more than 571 confirmed cases with 17 deaths had been reported in other parts of mainland China, and in various countries including South Korea, Japan, Thailand, Singapore, the Philippines, Mexico and the United States of America. As of 6 February 2020 (02:45 GMT), 28,276 cases, of which 3863 are in critical condition, and 565 deaths had been reported. 

The transmission potential, often measured in terms of the basic reproduction number, the outbreak peak time and value and duration under current and evolving intervention measures, remain unclear, and warrant further investigation. 

On 20 January 2020, the Chinese government revised the law provisions concerning infectious diseases to add the 2019-nCoV as a class B agent (a pathogen that can cause an epidemic outbreak). On the same day, public health officials announced a further revision to classify the novel virus as a class A agent (a pathogen that can cause an epidemic in a short time). Some non-pharmaceutical interventions (NPIs), including intensive contact tracing followed by quarantine of individuals potentially exposed to the disease, and isolation of infected, symptomatic individuals, were implemented, but their effectiveness during the early stage is questionable. 

Quantifying the effectiveness of these interventions is of crucial importance for Wuhan as well as for other cities in their preparedness and rapid response to the importation of infected cases. With the arrival of the Spring Festival, massive traveling is expected to mobilize a large segment of the population, by which the novel coronavirus may be broadly reseeded. 

Extreme, unprecedented measures have been taken. For example, on 23 January 2020, the Chinese authorities introduced travel restrictions affecting five cities (Wuhan, Huanggang, Ezhou, Chibi and Zhijiang), effectively shutting down the movement of more than 40 million people. 

However, how these expensive and resource-intensive measures can contribute to the prevention and control of the infection in these cities and other parts of the country, and how long these travel restrictions should be maintained, remain to be determined. In the context of a novel coronavirus affecting a naïve population, estimation of the basic reproduction number is important for determining the potential and severity of an outbreak, and providing critical information for designing and implementing disease outbreak responses in terms of the identification of the most appropriate, evidence-based interventions, mitigation measures and the determination of the intensity of such programs in order to achieve the maximal protection of the population with the minimal interruption of social-economic activities [8]. 

As recognized by the WHO [13], mathematical models, especially those which are timely, play a key role in informing evidence-based decisions by health decision- and policy-makers. To the best of our knowledge, only a few mathematical models have so far been publicly released, including a Bats-Hosts-Reservoir-People transmission network model and a returning traveler study aimed to compute underestimated coronavirus cases [14,15]. 

No study has focused on the practical implications of public health interventions and measures. Therefore, the present study was undertaken to fill in this gap of knowledge.

## 2. Experimental Section

### 2.1. Data

**Figure 1:**
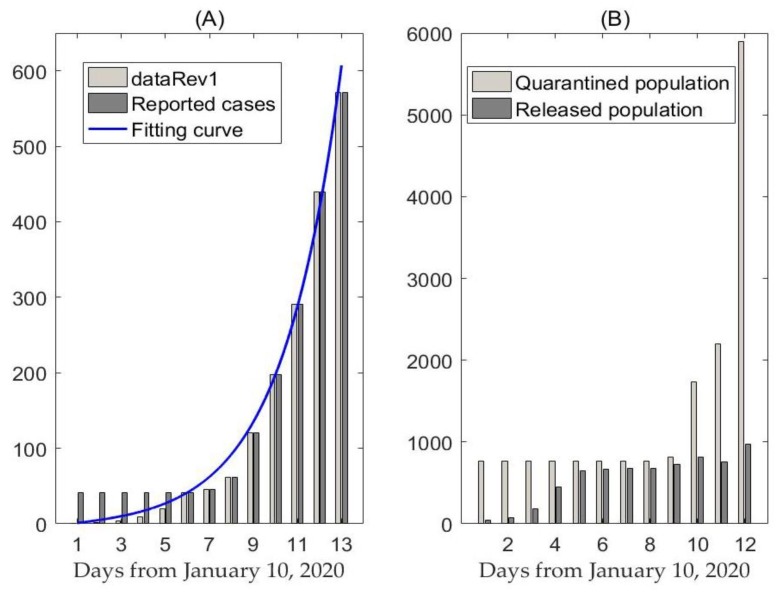
(**A**) Cumulative diagnoses and revised case data (dataRev1) in mainland China, the blue curve is the best fitting curve of model (1) to dataRev1. (**B**) Data information of cumulative quarantined/released population.

We obtained data of laboratory-confirmed 2019-nCoV cases which occurred in mainland China from the WHO situation report, the National Health Commission of the People’s Republic of China and the Health Commission of Wuhan City and Hubei Province [16,17,18,19]. Data information includes the cumulative number of reported cases, as shown in Figure 1A, and the quarantined and released population, as shown in Figure 1B. The data were released and analyzed anonymously. Since the identification of the 2019-nCoV on 10 January 2020, some cases were ruled out and the cumulative number of reported cases per day was 41, from 10 to 15 January 2020. To obtain the relatively reliable data, we used the exponential growth law to deduce the number of reported cases per day from 31 December 2019 to 10 January 2020 (called dataRev2) or from 10 to 15 January 2020 (called dataRev1) based on the 41 cases on that date, as shown in Figure 1A. 

By inferring the effectiveness of intervention measures, including quarantine and isolation (Figure 1B), we estimated the required effectiveness of these interventions in order to prevent the outbreak.

### 2.2. The Model

**Figure 2:**
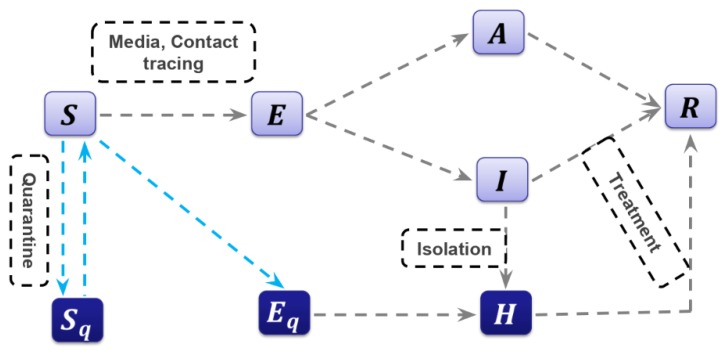
Diagram of the model adopted in the study for simulating the novel coronavirus (2019-nCoV) infection. Interventions including intensive contact tracing followed by quarantine and isolation are indicated.

Here, we propose a deterministic “Susceptible-Exposed-Infectious-Recovered” (SEIR) compartmental model based on the clinical progression of the disease, epidemiological status of the individuals and intervention measures (Figure 2). We parameterized the model using data obtained for the confirmed cases of 2019-nCoV in mainland China and estimated the basic reproduction number of the disease transmission.

In more detail, we investigated a general SEIR-type epidemiological model, which incorporates appropriate compartments relevant to interventions such as quarantine, isolation and treatment. We stratified the populations as susceptible (*S*), exposed (*E*), infectious but not yet symptomatic (pre-symptomatic) (*A*), infectious with symptoms (*I*), hospitalized (*H*) and recovered (*R*) compartments, and further stratified the population to include quarantined susceptible (*S_q_*), isolated exposed (*E_q_*) and isolated infected (*I_q_*) compartments. 

With contact tracing, a proportion, *q*, of individuals exposed to the virus is quarantined. The quarantined individuals can either move to the compartment *E_q_* or *S_q_*, depending on whether they are effectively infected or not [20], while the other proportion, 1 − *q*, consists of individuals exposed to the virus who are missed from the contact tracing and move to the exposed compartment, *E*, once effectively infected or stay in compartment *S* otherwise. Let the transmission probability be β and the contact rate be constant c. Then, the quarantined individuals, if infected (or uninfected), move to the compartment *E_q_* (or *S_q_*) at a rate of βc*q* (or (1 − β)c*q*). Those who are not quarantined, if infected, will move to the compartment *E* at a rate of βc(1−q). The infected individuals can be detected and then isolated at a rate $d_{I}$ and can also move to the compartment *R* due to recovery. 

The transmission dynamics are governed by the following system of equations:

(1) $$\begin{array}{l} S^{\prime }=-\left(\beta c+cq\left(1-\beta \right)\right)S\left(I+\theta A\right)+\lambda S_{q},\\ E^{\prime }=\beta c\left(1-q\right)S\left(I+\theta A\right)-\sigma E,\\ I^{\prime }=\sigma \varrho E-\left(\delta_{I}+\alpha +\gamma_{I}\right)I,\\ A^{\prime }=\sigma \left(1-\varrho \right)E-\gamma_{A}A,\\ S_{q}{}^{\prime }=\left(1-\beta \right)cqS\left(I+\theta A\right)-\lambda S_{q},\\ E_{q}{}^{\prime }=\beta cqS\left(I+\theta A\right)-\delta_{q}E_{q},\\ H^{\prime }=\delta_{I}I+\delta_{q}E_{q}-\left(\alpha +\gamma_{H}\right)H,\\ R^{\prime }=\gamma_{I}I+\gamma_{A}A+\gamma_{H}H, \end{array}$$

where ${}^{\prime }$ is the derivative with respect to time, and the other parameters are summarized in Table 1. 

### 2.3. Model-Based Method for Estimation

Given the model structure with quarantine and isolation (Figure 2), we used the next generation matrix [21,22] to derive a formula for the control reproduction number when control measures are in force, as follows:

(2) $$R_{c}=\left[\frac{\beta \varrho c\left(1-q\right)}{\delta_{I}+\alpha +\gamma_{I}}+\frac{\beta c\theta \left(1-\varrho \right)\left(1-q\right)}{\gamma_{A}}\right]S_{0}$$

We used the Markov Chain Monte Carlo (MCMC) method to fit the model and adopted an adaptive Metropolis–Hastings (M-H) algorithm to carry out the MCMC procedure. The algorithm is run for 100,000 iterations with a burn-in of the first 70,000 iterations, and the Geweke convergence diagnostic method is employed to assess convergence of chains.

### 2.4. Likelihood-Based Method for Estimation

We employed the likelihood-based method or generation interval-informed method of White and Pagano [23], using the following formula:

(3) $$L(R_{c},\;p|N)=\overset{T}{\underset{t=1}{\prod }}\frac{\exp\left(-\phi_{t}\right)\phi_{t}^{N_{t}}}{\Gamma \left(N_{t}+1\right)}$$

where $\phi_{t}=R_{c}\sum_{j=1}^{k}p_{j}N_{t-j}$, *k* is the maximum value of the serial interval (chosen as k=6 here) and Γ(x) is the gamma function. $N=\left\{N_{0},N_{1},\;\ldots \;,\;N_{T}\right\},$ where $N_{j}$ denotes the total number of cases on day j and T is the last day of observations. $p_{j}$ is the probability function for the generation interval on day j. We assume that the generation interval follows a gamma distribution with mean E and variance V. Since the generation interval of the 2019-nCoV is undetermined, we investigated the sensitivity of $R_{c}$ to different E values ranging from 2 to 8 days (given in Table 2). 

### 2.5. Simulation

The population of Wuhan is around 11,081,000 inhabitants [18], hence, we set S(0)=11,081,000. As of 10 January 2020, two patients had been recovered and were subsequently discharged from the hospital leading to R(0)=2, and 739 individuals were quarantined leading to $S_{q}\left(0\right)=739$. We set H(0)=1, corresponding to the reported confirmed case on 10 January 2020. The quarantined individuals were isolated for 14 days, thus λ=1/14. According to the WHO [24], the incubation period of 2019-nCoV is about 7 days, hence σ=1/7.

## 3. Results

### 3.1. Likelihood-Based Estimates

Likelihood-based estimation of $R_{c}$ during the outbreak in Wuhan gives a mean value of 6.39 with mean and variance of generation time of 6 and 2 days on the basis of a revised data series (dataRev1). The reproduction number based on likelihood-based estimation ranges from 1.66 to 10 and it follows from Table 2 that $R_{c}$ is sensitive to changes in mean generation intervals. Fitting to the other revised data series (dataRev2) gives a mean value of 6.32 with mean and variance of generation time of 6 and 2 days. Note that the estimates of $R_{c}$ based on the two time series agree well, and consequently, both revised data series can be used to fit the proposed dynamics transmission model. In this study, we chose the estimations based on dataRev1 as the comparison reference to verify and validate our model-based estimation. Thus, in the following sections of the manuscript, we will use the revised dataset (dataRev1) to fit the proposed model. 

### 3.2. Model-Based Estimates 

By fitting the model without considering asymptomatic infections to the data of hospital notification for the confirmed 2019-nCoV cases (dataRev1), we estimated the mean control reproductive number $R_{c}$ to be 6.47 (95% CI 5.71–7.23), whereas other parameter estimations are reported in Table 1. Note that the mean estimations of $R_{c}$ based on the likelihood method are within the 95% confidence interval of the model-based estimates (Table 2). 

Using the estimated parameter values, we predicted the trend of the 2019-nCoV infection. Under the current intervention (before 22 January 2020), the number of infected individuals (*I(t)*) is expected to peak on around 10 March 2020, with a peak size of $1.63\times 10^{5}$ infectious individuals. 

**Figure 3:**
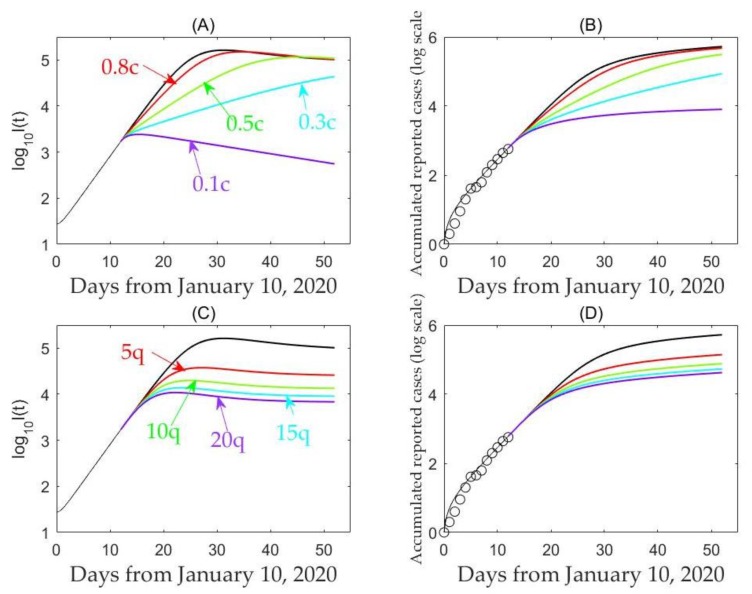
Sensitivity analyses with respect to contact rate, *c* (**A**,**B**), and quarantine rate, *q* (**C**,**D**), on the log number of infected individuals and cumulative reported cases.

To examine the possible impact of enhanced interventions on disease infections, we plotted the number of infected individuals (*I(t)*) and the predicted cumulative number of reported cases with varying quarantine rate q and contact rate c. This analysis shows that reducing the contact rate persistently decreases the peak value but may either delay or bring forward the peak, as shown in Figure 3 and Table 3. 

**Figure 4:**
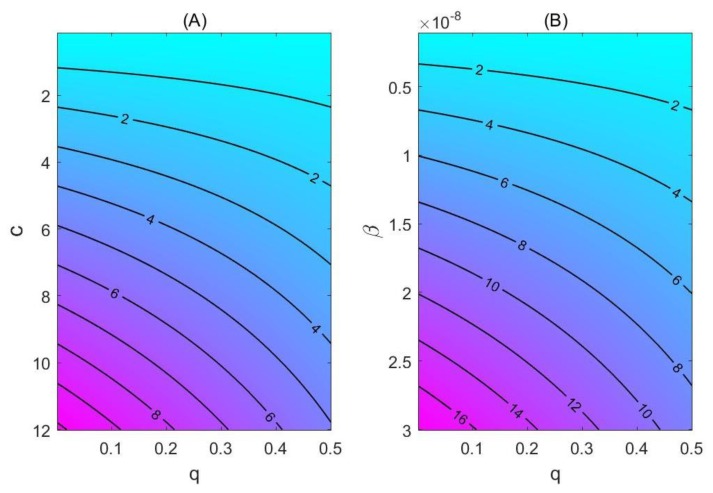
Contour plot of R_c, with the parameter of baseline transmission probability and the contact rate, *c* (**A**), or the quarantine rate, *q* (**B**). (**B**) shows that a higher transmission probability of the virus will significantly increase the basic reproduction number.

In more detail, our analysis shows that increasing quarantine rate, *q*, by 10 or 20 times will bring forward the peak by 6.5 or 9 days, and lead to a reduction of the peak value in terms of the number of infected individuals by 87% or 93%. This indicates that enhancing quarantine and isolation following contact tracing and reducing the contact rate can significantly lower the peak and reduce the cumulative number of predicted reported cases (Figure 4).

**Figure 5:**
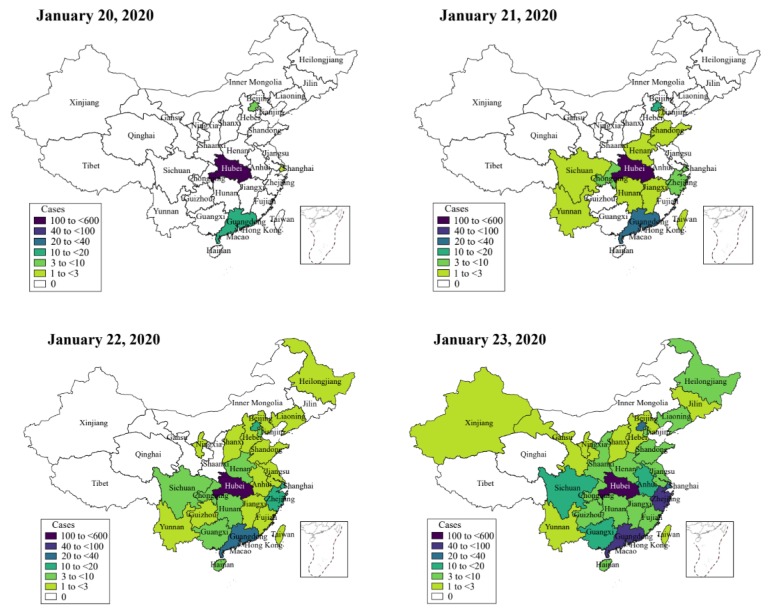
Heat-map showing the spreading of the Coronavirus infection.

Considering the spreading of the virus (Figure 5), and in order to examine the impact of the travel restriction on the infection in other cities such as Beijing, we initially calculated the daily number of exposed individuals imported from Wuhan to Beijing, denoted by Ime(t). 

According to our model, we get the exposed fraction as of 22 January 2020: approximately 40,000 persons from Wuhan to Beijing via trains (around 37,000) and flights (around 3000) [25], then, we have:

*Ime*(*t*) = 40,000 ∙ *E*(*t*)/*N* (4)

with 40 individuals being imported exposed individuals as of 22 January 2020. However, there could potentially exist an ascertainment bias in reported case data, since cases may have been larger than 40 individuals but have not been reported or reported with a delay in time.

**Figure 6:**
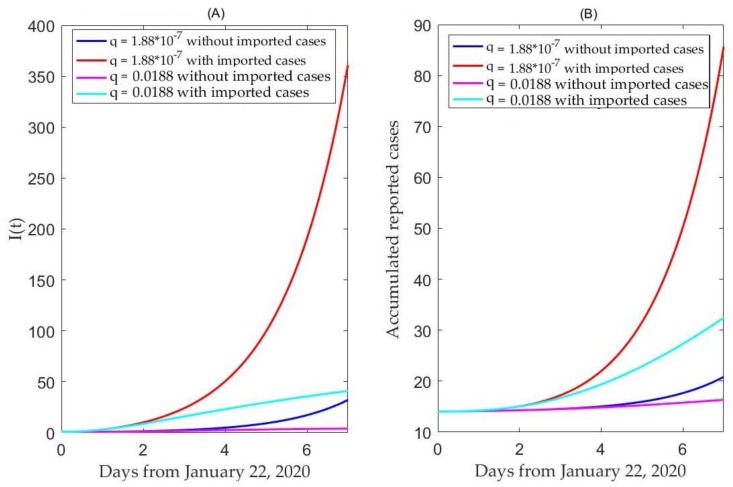
The effects of no travel restrictions (**A**) versus travel restriction (**B**) in the Hubei Province on the Coronavirus disease in Beijing city.

We find that with travel restriction (no imported exposed individuals to Beijing), the number of infected individuals in seven days will decrease by 91.14% in Beijing, compared with the scenario of no travel restriction, while, given no travel restriction, the number of infected individuals in seven days will decrease by 88.84% only if we increase the quarantine rate by 100 thousand times, as shown in Figure 6A. This means that the effect of a travel restriction in Wuhan on the 2019-nCoV infection in Beijing is almost equivalent to increasing quarantine by a 100 thousand baseline value, which is a rate that can hardly be achieved in any public health setting. It follows from Figure 6B that with travel restriction, the number of cumulative individuals in seven days will significantly decrease (by 75.70%) in Beijing, compared with the scenario of no travel restriction. 

## 4. Discussion

Based on the 2019-nCoV cases’ data until 22 January 2020, we have estimated the basic reproduction numbers using different methods (likelihood-based and model-based approaches). The mean control reproduction number was estimated to be as high as 6.47 (95% CI 5.71–7.23), in comparison with the values of the SARS epidemics (R_0_ = 4.91) in Beijing, China, in 2003 [26], and MERS in Jeddah (R_0_ = 3.5–6.7) and Riyadh (R_0_ = 2.0–2.8), Kingdom of Saudi Arabia, in 2014 [27]. 

Our value is higher than other published estimates (for instance, Reference [28]). Such a high reproduction number is consistent with the opinion that the virus has gone through at least three–four generations of transmission in the period covered by this study [24]. Note that our estimation is based on a dataset collected during a period of intensive social contacts. Before the Chinese New Year (25 January 2020), there were lots of annual summing-up meetings and/or parties, with higher than usual close contacts, leading to a higher likelihood of infection transmission than that of the earlier periods covered by other studies. Furthermore, we noted that more recently published studies based on datasets during periods comparable with ours reported similar findings in terms of a high basic reproduction number (for instance, Reference [29], where authors, using an exponential growth method, computed a basic reproduction number of 6.11 (95% CI 4.51–8.16), assuming no changes in reporting rate and with a serial interval of 8.4 ± 3.8 days). Variability in the estimation of the basic reproduction number is also a well-known methodological issue, and standardized methods both for calculating and reporting it are still lacking [30]. During the initial phases of an epidemics outbreak, only small datasets/time-points can be used. Some crucial information may be missing, and the quality, accuracy and reliability of data improves over time. In these situations, estimations are highly dependent on the specific datasets utilized and revising/updating such datasets could influence the results. We note that several key clinical parameters could be inferred from relevant clinical data based on sero-epidemiological surveys, and the possibility of spreading the infection from asymptomatic cases was only reported recently [31].

Our finding of a high reproduction number implies the potential of a very serious epidemic unless rather swift public health interventions are implemented [32,33], during the season when the social contacts is the highest. 

Note that the serial interval is an essential factor affecting the accuracy of the likelihood function estimation. According to the current report, the incubation period of Wuhan patients with coronavirus pneumonia is about 2 to 15 days. We then assume that the serial interval follows the gamma distribution with varying mean and variance, which allows us to examine the influence on the reproduction number. With the distribution of serial interval with mean 6 days and variance 2 days, the likelihood-based estimation of the reproduction number is consistent with the model-based estimation. It shows that longer serial intervals induce greater reproduction numbers, and hence, more new infections, which further confirms that the epidemic may be more serious than what has been reported until now [15]. 

Based on the reported data, we have estimated that the number of people who were identified through contact tracing and quarantined was 5897, as of 22 January 2020. In comparison with the total population size of Wuhan, the effort of close contact tracing and quarantine was insufficient and appears to have a limited impact in terms of reducing the number of infected cases and/or slowing down the epidemic. The contour plot of R_c = 1 gives the threshold values of contact rate and quarantine rate for a city to avoid an outbreak. This high threshold rate of quarantine puts an extremely high requirement for the city’s public health infrastructure and its citizens’ adherence to personal protective and public health interventions, including a reduction of transmission-effective contacts, separation and restriction during the quarantine. 

Such a high level of quarantine rate and reduction of contact is possible only when the number of imported cases from the epicenter is minimal, speaking in terms of the value of the travel restriction. A strict travel restriction to the city of Wuhan is expensive and resource-consuming, imposing a substantial challenge to the decision- and policy-makers and the city’s resilience. Moreover, such a measure could only delay the transmission of the infectious disorder. 

In conclusion, our simulations show that the appropriate duration of this travel restriction depends on a combination of effective quarantine and reduction of contact within the city.

Considering the latest events (the lock-down of Wuhan on 23 January 2020, the adoption of the travel restriction strategy by other regions and provinces, the introduction of new detection technologies, etc.), the present model needs to be revised in that the basic reproduction number estimated here is no longer suitable for predicting future epidemic trends (Table 4). This will be the aim of a forthcoming article.

## 5. Conclusions

Coronaviruses occasionally lead to major outbreaks, with documented reproduction numbers ranging from 2.0 to 4.9. Currently, a fourth large-scale outbreak is occurring and spreading out from Wuhan, Hubei province, China, to neighboring provinces and other countries. There is a dearth of epidemiological data about the emerging coronavirus, which would be of crucial importance to design and implement timely, ad hoc effective public health interventions, such as contact tracing, quarantine and travel restrictions. In this study, we adopted a deterministic model to shed light on the transmission dynamics of the novel coronavirus and assess the impact of public health interventions on infection. We found that the basic reproduction number could be as high as 6.47 (95% CI 5.71–7.23), which seems consistent with the special period prior to the Spring Festival when contacts were higher than usual, and with the opinion that the virus has gone through at least three–four generations. It is worth mentioning that our model made a very good prediction of the confirmed cases from 23 to 29 January 2020, as shown in Table 4. Particularly, the predicted confirmed cases should be 7723 as of 29 January 2020, which is very close to the real number of cases of 7711. Furthermore, according to our model, the outbreak, under the most restrictive measures, is expected to peak within two weeks (since 23 January 2020), with a significant low peak value. Our investigation has major practical implications for public health decision- and policy-makers. The rather high reproduction number suggests that the outbreak may be more serious than what has been reported so far, given the particular season of increasing social contacts, warranting effective, strict public health measures aimed to mitigate the burden generated by the spreading of the new virus.

## Figures and Tables

**Table 1:** Parameter estimates for 2019-nCoV in Wuhan, China.

**Parameter**	**Definitions**	**Estimated Mean Value**	**Standard Deviation**	**Data Source**
c	Contact rate	14.781	0.904	MCMC
β	Probability of transmission per contact	$2.1011\times 10^{-8}$	$1.1886\times 10^{-9}$	MCMC
q	Quarantined rate of exposed individuals	$1.8887\times 10^{-7}$	$6.3654\times 10^{-8}$	MCMC
σ	Transition rate of exposed individuals to the infected class	1/7	–	WHO
λ	Rate at which the quarantined uninfected contacts were released into the wider community	1/14	–	[18,19]
ϱ	Probability of having symptoms among infected individuals	0.86834	0.049227	MCMC
$\delta_{I}$	Transition rate of symptomatic infected individuals to the quarantined infected class	0.13266	0.021315	MCMC
$\delta_{q}$	Transition rate of quarantined exposed individuals to the quarantined infected class	0.1259	0.052032	MCMC
$\gamma_{I}$	Recovery rate of symptomatic infected individuals	0.33029	0.052135	MCMC
$\gamma_{A}$	Recovery rate of asymptomatic infected individuals	0.13978	0.034821	MCMC
$\gamma_{H}$	Recovery rate of quarantined infected individuals	0.11624	0.038725	MCMC
α	Disease-induced death rate	$1.7826\times 10^{-5}$	$6.8331\times 10^{-6}$	MCMC
**Initial Values**	**Definitions**	**Estimated Mean Value**	**Standard Deviation**	**Data Source**
S(0)	Initial susceptible population	11,081,000	–	[18]
E(0)	Initial exposed population	105.1	35.465	MCMC
I(0)	Initial symptomatic infected population	27.679	11.551	MCMC
A(0)	Initial asymptomatic infected population	53.839	25.25	MCMC
$S_{q}\left(0\right)$	Initial quarantined susceptible population	739	–	[18]
$E_{q}\left(0\right)$	Initial quarantined exposed population	1.1642	0.20778	MCMC
H(0)	Initial quarantined infected population	1	–	[18]
R(0)	Initial recovered population	2	–	[18]

Markov Chain Monte Carlo (MCMC); World Health Organization (WHO).

**Table 2:** Estimation of the basic reproduction number for 2019-nCoV in Wuhan, China.

R_0_	V = 2 (dataRev1)	V = 3 (dataRev1)	V = 2 (dataRev2)	V = 3 (dataRev2)
E = 2	1.4546	1.6560	1.4545	1.6554
E = 3	1.7459	1.7155	1.7456	1.7145
E = 4	2.5828	2.4462	2.5815	2.4427
E = 5	3.9893	3.7134	3.9802	3.6956
E = 6	6.3901	5.8303	6.3164	5.7304
E = 7	10	9.2564	9.6409	8.7299
E = 8	10	10	10	10

E: mean value of Gamma distribution, V: Deviation of Gamma distribution dataRev1: [1 2 3 4 6 9 12 20 28 41 45 62 121 198 291 440 571]; dataRev2: [1 2 3 4 6 9 12 20 28 41 41 41 41 41 41 45 62 121 198 291 440 571].

**Table 3:** The effects of travel restrictions on the peak time and peak value.

Parameter c	c	0.8c	0.5c	0.3c	0.1c
Peak Time	19.3 days	22.6 days	33.8 days	61.3 days	3.4 days
Value of I at peak time	$1.63\times 10^{5}$	$1.5\times 10^{5}$	$1.15\times 10^{5}$	$6.68\times 10^{4}$	$2.42\times 10^{3}$
Parameter q	q	5q	10q	15q	20q
Peak time	19.3 days	15.1 days	12.8 days	11.4 days	10.3 days
Value of I at peak time	$1.63\times 10^{5}$	$3.76\times 10^{4}$	$1.98\times 10^{4}$	$1.38\times 10^{4}$	$1.08\times 10^{4}$

Note that the baseline values are (*c, q*) = (14.78, 1.88 × 10^−7^).

**Table 4:** Predictions of the confirmed cases.

Date	01/23	01/24	01/25	01/26	01/27	01/28	01/29
Predicted confirmed cases	876	1266	1828	2634	3784	5419	7723
Predicted confirmed cases (reduced contact by 50%)	868	1207	1624	2128	2736	3464	4335
Predicted confirmed cases (reduced contacts by 90%)	862	1163	1480	1802	2120	2430	2731
Real data of confirmed cases	830	1287	1975	2744	4515	5974	7711
